# Unusual Life-Threatening Complication of a Substernal Goiter: A Case of an Upper Gastrointestinal Bleed

**DOI:** 10.7759/cureus.34633

**Published:** 2023-02-04

**Authors:** Maria Leonor Guia Lopes, Catarina O'neill, Carolina Antunes, Clotilde Limbert, João Sequeira Duarte

**Affiliations:** 1 Endocrinology Department, Hospital Egas Moniz - Centro Hospitalar de Lisboa Ocidental, Lisboa, PRT; 2 Gastroenterology Department, Hospital Egas Moniz - Centro Hospitalar de Lisboa Ocidental, Lisboa, PRT

**Keywords:** emergency gastroenterology and endoscopy, superior vena cava (svc) syndrome, thyroid volume, esophageal variceal bleed (evb), substernal goiter

## Abstract

Substernal goiter represents a common and challenging clinical scenario in medical practice. Symptoms often include dysphagia, dyspnea, and hoarseness, deeming the vascular compressive symptoms an unusual finding. In extraordinarily rare cases, its slow and gradual growth determines the emergence of severe superior vena cava syndrome, with consequent development of upper esophageal downhill varices. In contrast with distal esophageal varices, downhill variceal hemorrhage is extremely rare.

The authors report a patient admitted to the emergency room due to upper gastrointestinal hemorrhage, caused by downhill upper esophageal varices’ rupture, secondary to compressive substernal goiter.

In this case, irregular follow-up resulted in massive thyroid growth, progressive vascular and airway compression, and the development of venous collateral pathways. Despite the severity of those compressive symptoms, the patient was not a surgical candidate considering her multiple cardiovascular and respiratory comorbidities. Newly developed thyroid ablative techniques may emerge as a possible life-saving treatment when the surgical approach cannot be considered.

## Introduction

Thyroid goiter growth may prompt its presence in the thoracic inlet, causing the compression of its structures, and being defined as substernal or dipping goiter. In these cases, cervical and thoracic computed tomographies (CT) are the best diagnostic imaging tools, with high attenuation on non-contrast-enhanced images, and marked enhancement after intravenous contrast [1,2].

In very rare cases, the dimensions of the intrathoracic immersing thyroid components can cause compression of cervicothoracic structures, mainly the tracheobronchial tree and major vascular branches [1,3].

Superior vena cava syndrome (SVCS) results from compression to the venous drainage caused most commonly by malignant masses (ganglionic, mediastinal, or bronchopulmonary) [3]. The most common symptoms of SVCS are dyspnoea, facial congestion, venous distension of the chest wall, arm swelling, and facial plethora [4].

Substernal benign goiter is an infrequent cause of SVCS. In such cases, patients may be asymptomatic due to venous collateral development during the slow growth of the thyroid parenchyma, resulting in a delay in the diagnosis. Furthermore, the consequent collateral circulation, secondary to SVCS, can be found in the upper or middle third of the esophageal mucosa, resulting in downhill esophageal varices. In contrast to the usual lower esophageal varices, usually a consequence of portal hypertension, downhill varices develop in the deep submucosa layer. Downhill variceal bleeding is extremely rare, accounting for 0.1% of patients presenting with haematemesis [5].

## Case presentation

An 81-year-old woman was admitted to the emergency room (ER) presenting melenas, asthenia, and severe fatigue. Her relevant long-term medical history consisted of chronic obstructive pulmonary disease (COPD), arterial hypertension, and severe heart failure (NYHA III). Moreover, 13 years before the present admission, the patient had been diagnosed with multinodular substernal goiter. At that time, cervical and thoracic CT reported a global enlargement of the thyroid gland without compression of the airway or vascular thoracic structures. Bilateral cytologic results revealed a benign thyroid hyperplasia. She refused any attempt of surgical correction, despite physician counseling about the accompanying potential life-threatening risks. 

First physical examination at the ER revealed a hemodynamically unstable patient (systolic blood pressure of 86 mmHg, tachycardia of 111 bpm, and severe anemia) (Table 1), due to severe blood loss, requiring emergent fluid resuscitation and transfusional support. 

**Table 1:** Laboratory data at the admission to the Emergency department

Variable	Result	Reference Range
Haemoglobin (g/dL)	7.5	12.0-15.0
Red blood cells (×10^9^/L)	2.3	3.9-15.0
Mean corpuscular volume (fL)	95.0	80.0-96.1
Mean Globular Haemoglobin (pg)	32.9	27.3-33.7
Mean Corpuscular Haemoglobin concentration (MCHC) (g/L)	328.0	328.0-354.0
Red Cell Distribution Width (%)	16.3	11.5-14.5
Transferrin saturation (%)	46.0	20.0-45.0
Iron (μg/dL)	123.0	33.0-193.0
Ferritin (ng/mL)	83.2	30.0-340.0
Total iron-biding capacity - TIBC (μg/dL)	268.0	250.0-425.0
Folates (nmol/L)	16.1	10.0-42.0
B12 vitamin (pmol/L)	232.0	141.0-489.0
White blood cells (×10^9^/L)	12.8	4.0-10.0
Platelets (10^9^/L)	146.0	250.0-400.0
Creatinine (mg/dL)	2.1	0.5-0.9
Urea (mg/dL)	234.0	17.0-49.0
Aspartate aminotrasferase (U/L)	15	<32
Alanine aminotrasferase (U/L)	6	<33
Alkaline phosphatase (U/L)	60	35-104
Albumin (g/dL)	3.7	3.5-5.2
Potassium (mmol/L)	5.7	3.5-5.5
Prothrombin time (seconds)	11.9	<14.0
International normalised ratio - INR	1.1	1.0
Activated partial thromboplastin time - aPTT	22.2	23.0-38.0

**Figure 1:**
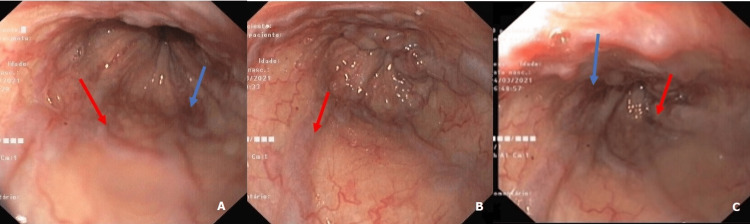
Upper endoscopic imaging (A - Cervical esophagus; B and C - Upper and middle thoracic esophagus) revealing downhill varices (red arrows), mucosal hyperemia and red spots (blue arrows) suggesting recent bleeding.

Considering the severity of the patient’s clinical state, an emergent upper endoscopy was performed (Figure 1). A tortuous esophageal mucosa was evidenced along with bulging of the proximal esophageal segment and extensive collateral venous circulation (downhill varices). Red spots on these varices were also observed, suggesting recent hemorrhage without signs of active bleeding. 

**Figure 2:**
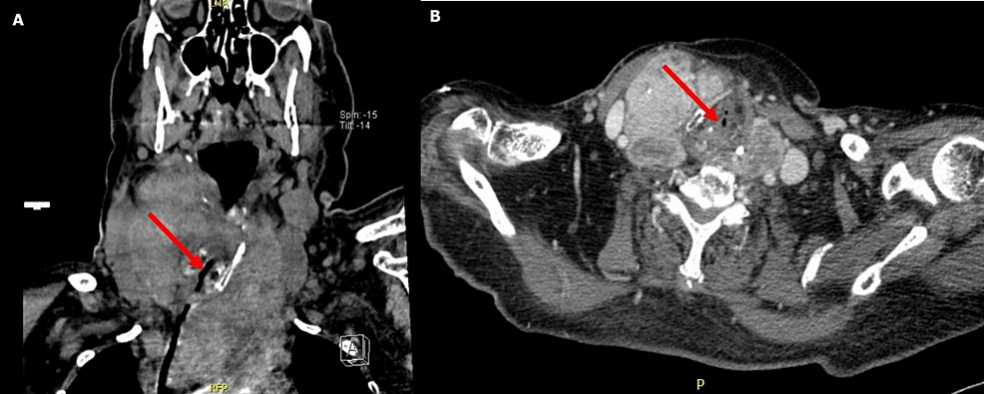
Neck and thoracic CT scan (coronal and axial) showing massive vascular and airway compression by substernal goitre. Trachea location is identified using red arrows.

Subsequent neck and thoracic evaluation, using CT scan imaging (Figure 2), documented a substernal asymmetric goiter (right lobe - 90x67x56 mm; left lobe 125x75x90 mm). The left dipping lobe reached the azygos vein crossing, causing compression of the trachea, esophagus, and both brachiocephalic veins. Moreover, the minimum registered trachea diameter was 7 millimeters.

At the time of the ER admission, the patient could not be considered a surgical candidate, due to a high anesthetic risk defined by cardiac and respiratory severe comorbidities (COPD, heart failure). Furthermore, the patient refused local treatment with endoscopic elastic band ligation, as well as other therapeutic options such as thyroid ablative techniques. The patient died two weeks after hospital discharge.

## Discussion

The authors report a rare case of a patient presenting with downhill variceal hemorrhage secondary to SVCS, as a result of compressive substernal benign goiter. In this particular case, the progressive tissue growth over time determined the presented aggressive structural compression. 

Downhill varices, first described in 1964, are located in the upper third of the esophagus and provide an alternative route for venous return when systemic venous blood flow is obstructed. In contrast to inferior esophageal varices, downhill varices are extremely rare causes of gastrointestinal bleeding [3,5-7]. Several factors can be accounted for this lower hemorrhagic risk: the negligible exposure to gastric acid, the absence of coagulation cascade disruption (when comparing with varices resulting from portal hypertension due to hepatic failure), and the submucosal deep location of downhill varices (in contrast to the more superficial location of lower third esophagus varices) [3,6]. 

Currently, there are no international recommendations on screening or management of downhill varices. Upper endoscopy should be considered in these cases considering that it allows direct visualization of proximal varices along with local intervention when clinically appropiate [7]. 

Therefore, similarly to other gastrointestinal bleeding clinical events, the treatment of downhill varices hemorrhage depends on its hemodynamical and analytical repercussions. That may rely on emergent endoscopic techniques, such as the Sengstaken-Blakemore balloon, local coagulation, or elastic banding. However, octreotide, as a splanchnic dilator, is not expected to effectively decrease the pressure in the upper esophageal varices as they do not communicate directly with the portal system [8].

In what concerns the rare cases of substernal goiter as the cause of SVCS and downhill varices, thyroid surgical resection is usually the proposed definitive therapy. In 2020, Papini et al. proposed the use of image-guided thermal ablation (TA) as a cost- and risk-effective alternative in adult patients with benign thyroid nodules with compressive symptoms who decline surgery [9]. These newly developed thyroid ablative techniques may, therefore, emerge as a possible life-saving treatment when the surgical approach can not be considered.

## Conclusions

The authors report a rare case of a patient admitted to the emergency room due to upper gastrointestinal hemorrhage, caused by downhill upper esophageal varices’ rupture, secondary to compressive substernal goiter.

In this particular case, early surgical therapy could have potentially prevented the clinical outcome. On the other hand, the use of new ablative techniques, such as radiofrequency or laser, which the patient declined, could have been performed to increase patient's quality of life. 
